# The effect of smoking on biological change of recurrent breast cancer

**DOI:** 10.1186/s12967-020-02307-x

**Published:** 2020-04-05

**Authors:** Koji Takada, Shinichiro Kashiwagi, Yuka Asano, Wataru Goto, Rika Kouhashi, Akimichi Yabumoto, Tamami Morisaki, Hisakazu Fujita, Masatsune Shibutani, Tsutomu Takashima, Kosei Hirakawa, Masaichi Ohira

**Affiliations:** 1 Department of Breast and Endocrine Surgery, Osaka City University Graduate School of Medicine, 1-4-3 Asahi-machi, Abeno-ku, Osaka, 545-8585 Japan; 2 Department of Scientific and Linguistic Fundamentals of Nursing, Osaka City University Graduate School of Nursing, 1-5-17 Asahi-machi, Abeno-ku, Osaka, 545-0051 Japan; 3 Department of Gastrointestinal Surgery, Osaka City University Graduate School of Medicine, 1-4-3 Asahi-machi, Abeno-ku, Osaka, 545-8585 Japan

**Keywords:** Recurrent breast cancer, Smoking, HER2, Pack-year, Tobacco

## Abstract

**Background:**

The selection of treatment for a patient with breast cancer largely relies on the cancer subtype. However, this process is complicated by changes in tumor biology at relapse. Smoking has been identified as a risk factor for breast cancer. The direct effect of a tobacco component delivered via blood circulation on the mammary gland tissue and subsequent DNA damage have been proposed to explain the association between cigarette smoking and breast cancer carcinogenesis. This postulation is supported by both tissue culture and animal studies demonstrating that the associated DNA damage further alters breast cancer cells, as indicated by an increased proliferative capacity and malignant transformation. In this study, we aimed to explore the relationship between changes in Estrogen receptor (ER), progesterone receptor (PgR), and human epidermal growth factor receptor 2 (HER2) each receptor at recurrence, and smoking and the prognosis after recurrence.

**Methods:**

This retrospective study included 989 patients with primary breast cancer who developed relapse after surgery and 50 patients who underwent regenerative biopsy or surgery from December 2007 to March 2018. ER, PgR, and HER2 expression in the primary and recurrent lesions was evaluated using immunohistochemistry, and the correlations of these expression patterns with smoking history (pack-years) were examined.

**Results:**

When ER was evaluated in recurrent tumors, negative and positive conversions were recognized in 3 (6.0%) and 1 patient (2.0%), respectively. When PgR was evaluated, negative conversion was recognized in 15 patients (30.0%). When HER2 was evaluated, positive conversion was recognized in 6 patients (12.0%). Consequently, we observed a change in the intrinsic subtype in in 5 patients with recurrent tumors (10.0%). Although most clinical factors were not correlated with smoking, a positive conversion of HER2 in recurrence was significantly more frequent among smokers than among non-smokers (p = 0.024).

**Conclusions:**

Biological changes during breast cancer recurrence should be given careful clinical consideration because they affect treatment after recurrence. Our results suggest that smoking may induce increased HER2 expression in recurrent breast tumors.

## Background

Smoking is a risk factor for the development of breast cancer [1]. One proposed explanation for this link between cigarettes and breast cancer carcinogenesis suggests that a tobacco component is delivered directly to the mammary gland tissue via blood circulation, leading to DNA damage in the mammary gland cells [2, 3]. This potential mechanism is supported by tissue culture and animal experiments in which this damage causes changes in breast cells, such as an increased proliferative capacity and malignant transformation [4–6].

When determining the course of breast cancer treatment, it is important to evaluate the status of estrogen receptor (ER), progesterone receptor (PgR), and human epidermal growth factor receptor 2 (HER2) expression. However, changes in the receptor expression patterns over the course of treatment can present clinical challenges. Specifically, these patterns often differ between primary and recurrent tumors, leading to a poor prognosis after recurrence [7, 8]. Therefore, it is necessary to re-evaluate the receptor expression status when a recurrent tumor arises. Given the potential effects of tobacco components on breast cancer cell traits, we hypothesized that smoking may contribute to these changes in receptor expression in recurrent disease. In this study, we aimed to analyze the relationships between changes in each receptor at recurrence, smoking and the subsequent prognosis.

## Methods

### Patient background

This retrospective study included 989 patients with resectable primary breast cancer who underwent curative resection as the first-line treatment between December 2007 and March 2018 at the Osaka City University Hospital (Osaka, Japan). Patients who received preoperative treatment and those with synchronous or metachronous bilateral breast cancer cases were excluded. At this institution, the patient’s smoking history (cigarettes smoked per day and years of smoking) is routinely recorded at the first visit, which yields the data necessary to calculate the pack-years as the number of cigarettes smoked per day divided by 20, then multiplied by the number of smoking years.

Each breast cancer received a definitive pathological diagnosis and was subjected to immunohistochemistry to determine the expression of ER, PgR, HER2, and Ki-67 (proliferation index). Based on the results, we classified the tumors in accordance with our previous work as hormone receptor-positive breast cancer (HRBC; ER- and/or PgR-positive), HER2-enriched breast cancer (HER2BC; ER−, PgR−, and HER2+), or triple-negative breast cancer (TNBC; ER−, PgR−, and HER2−) [9, 10]. We also applied a Ki-67 cutoff of 14% with reference to a previous report [11]. Tumor stage and resectability were evaluated using ultrasonography (US), computed tomography (CT), and bone scintigraphy.

Patients underwent primary tumor resection via mastectomy or breast-conserving surgery. Sentinel node biopsy or axillary dissection was performed in cases involving axillary nodal surgery; in the former cases, the detection of a sentinel node macrometastasis indicated the need for subsequent axillary dissection. After surgery, the patient was administered postoperative radiotherapy, delivered to the remnant breast, and standard postoperative adjuvant therapy according to the pathological diagnosis of the resected specimen. However, some patients did not receive postoperative treatment because of refusal or a poor general condition. All patients were followed-up via physical examinations, US, CT and bone scintigraphy according to the degree of recurrence risk.

**Fig. 1:**
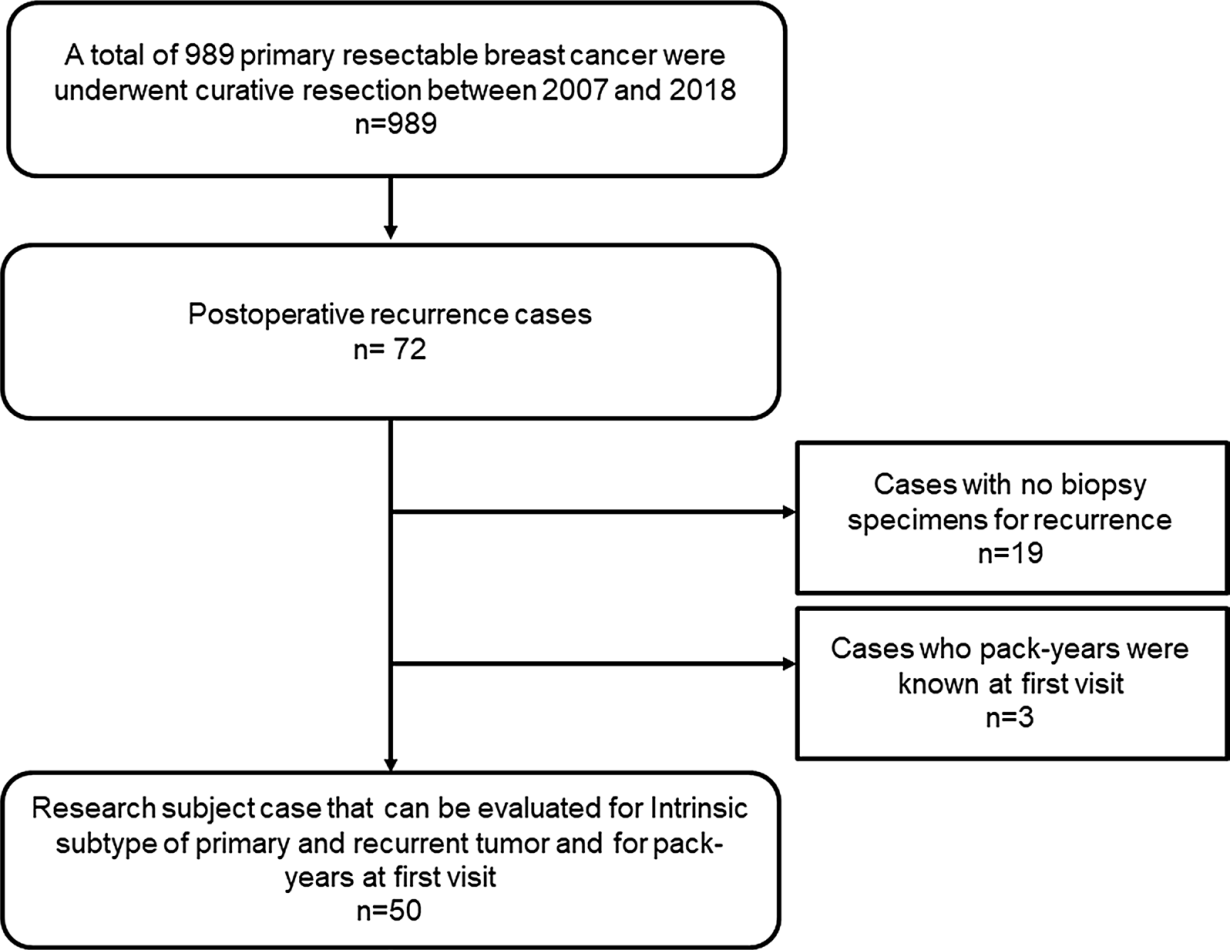
Consort diagram. Recurrence occurred in 77 of 989 patients who underwent curative resection. However, nineteen of them did not undergo biopsy because of distant metastatic recurrence. In this study, such cases are also included. Of the remaining, 3 patients did not know the smoking history, so we studied in the remaining 50 cases

Recurrence occurred in 77 of 989 patients who underwent curative resection. However, 19 of these patients did not undergo biopsy because the recurrent disease involved distant metastasis. This study also included some cases of distant metastasis wherein a biopsy was performed because it was difficult to differentiate the primary cancer of another organ from a distant metastasis of breast cancer. Of the remaining patients with recurrent disease, smoking history were not available for 3 patients. Therefore, we studied the remaining 50 cases (Fig. 1), all of whom underwent biopsy or resection immediately after relapse. No biopsies or resections were performed after the administration of antitumor drug treatment for recurrent disease.

Regarding survival outcomes, progression-free survival (PFS) was defined as the time interval from recurrence to deterioration by treatment started after recurrence or death. Post-recurrence survival (PRS) was defined as the time interval from recurrence to death. The 50 patients with recurrent disease were followed for a median of 2128 days (range, 416-–3789 days) postoperatively.

### Statistical analysis

Comparisons between the two groups were performed using the Chi square test. The odds ratio (OR) and 95% confidence intervals (CI) were calculated by the logistic analysis. PFS and PRS were estimated using the Kaplan–Meier method and compared between groups using the log-rank test. The hazard ratios (HRs) and 95% confidence intervals (CIs) were calculated using the Cox proportional hazards model. A multivariable analysis was performed using the Cox regression model. All statistical analyses were performed using the JMP software package (SAS, Tokyo, Japan), and statistical significance was defined as a *p* value of < 0.05.

### Ethics statement

This study was conducted at the Osaka City University Graduate School of Medicine, according to the Reporting Recommendations for Tumor Marker Prognostic Studies (REMARK) guidelines. The study protocol involved a retrospectively written plan of research, pathological evaluation, and statistical analysis [12]. The study complied with the provisions of the Declaration of Helsinki, and all patients provided written informed consent for their treatment and data collection. The retrospective protocol was approved by the ethics committee of Osaka City University (approval number #926).

## Results

### Clinicopathological features

Fifty patients underwent radical surgery without preoperative treatment and a biopsy or resection of a recurrent tumor (Table 1). The median age of these patients was 60 (range, 37–79) years, and the median tumor size at the time of surgery was 21.8 (8.0–45.0). Fourteen patients (28.0%) had a history of smoking before surgery, with a median duration of 30 (1.4–150) pack-years. An evaluation of surgical specimens revealed that seven patients (14.0%) had lymph node metastases, as well as the following distribution of intrinsic subtypes: HRBC, 38 cases (76.0%); HER2BC, 2 (4.0%) cases; and TNBC, 10 (20.0%) cases. All patients with HRBC had a HER2-negative status. Thirteen patients (26.0%) with a pathological diagnosis that suggested a high risk of recurrence received postoperative adjuvant chemotherapy. Eleven patients (22.0%) received postoperative radiotherapy delivered to the remnant breast, and 1 patient (2.0%) received trastuzumab therapy. Moreover, 72% of all patients received endocrine therapy, and this high rate was attributed to the administration of this type of therapy to most patients with HRBC. In contrast, 6 patients (12.0%) did not receive any postoperative treatment.

**Table 1:** Clinicopathological features of 50 cases biopsied/resected to recurrence

Parameters	Number of patients (n = 50) (%)
Age at operation (years old)	60 (37–79)
Tumor size (mm)	21.8 (8.0–45.0)
Lymph node metastasis	
N0/N1/N2	43 (86.0%)/5 (10.0%)/2 (4.0%)
Estrogen receptor (ER) of primary tumor	
Negative/positive	13 (26.0%)/37 (74.0%)
Progesterone receptor (PgR) of primary tumor	
Negative/positive	15 (30.0%)/35 (70.0%)
HER2 of primary tumor	
Negative/positive	48 (96.0%)/2 (4.0%)
Ki67 of primary tumor	
≤14%/> 14%	19 (38.0%)/31 (62.0%)
Intrinsic subtype	
HRBC/HER2BC/TNBC	38 (76.0%)/2 (4.0%)/10 (20.0%)
Chemotherapy after surgery	
No/yes	37 (74.0%)/13 (26.0%)
Endocrine therapy after surgery	
No/yes	14 (28.0%)/36 (72.0%)
Radiation therapy after surgery	
No/yes	39 (78.0%)/11 (22.0%)
Trastuzumab after surgery	
No/yes	49 (98.0%)/1 (2.0%)
No-treatment after surgery	
No/yes	44 (88.0%)/6 (12.0%)
Age at recurrence (years old)	62 (41–86)
Recurrent tumor site biopsied	
Local/regional lymph node/lung/brain/liver	30 (60.0%)/15 (30.0%)/3 (6.0%)/1 (2.0%)/1 (2.0%)
Change in expression of ER in recurrent tumor	
Negative conversion/no change/positive conversion	3 (6.0%)/46 (92.0%)/1 (2.0%)
Change in expression of PgR in recurrent tumor	
Negative conversion/no change/positive conversion	15 (30.0%)/35 (70.0%)/0 (0.0%)
Change in expression of HER2 in recurrent tumor	
Negative conversion/no change/positive conversion	0 (0.0%)/44 (88.0%)/6 (12.0%)
Change of intrinsic subtype in recurrent tumor	
No/yes	45 (90.0%)/5 (10.0%)
Disease free survival	792 (99–3300)
Smoker	
No/yes	36 (72.0%)/14 (28.0%)
Pack-years of smoker	30 (1.4–150)

*HER2* human epidermal growth factor receptor 2, *HRBC* hormone receptor-positive breast cancer (ER+ and/or PgR+), *HER2BC* human epidermal growth factor receptor 2-enriched breast cancer (ER−, PgR−, and HER2+), *TNBC* triple negative breast cancer (ER−, PgR−, and HER2−)

The median DFS duration was 792 (99–3300) days, and the median age at recurrence was 62 (range 41–86) years. Most biopsied recurrent tumors involved the local or regional lymph nodes, although biopsies were obtained from distant metastases in 5 cases (10.0%), including the lung in 3 cases (6.0%), brain in 1 case (2.0%), and liver in 1 case (2.0%). In 7 patients (14.0%), recurrences were observed in organs that were not biopsied simultaneously (Additional file 1: Table S1), including 3 patients who underwent biopsy of a local recurrence and also presented with lymph node metastasis, lung metastasis, or bone metastasis and 4 patients who underwent biopsy of a regional lymph node recurrence who presented with lung metastasis. No cases involved simultaneous recurrent lesions in 3 or more organs.

We further explored receptor expression in the recurrent tumors via histopathology. Regarding ER, negative conversion was recognized in 3 patients (6.0%) and positive conversion was recognized in 1 patient (2.0%). Regarding PgR, negative conversion was recognized in 15 patients (30.0%). Regarding HER2, positive conversion was recognized in 6 patients (12.0%). Consequently, 5 patients (10.0%) exhibited a change of intrinsic subtype upon recurrence.

### Correlation between changes in receptor expression and clinical factors

The potential correlations of these receptor expression changes with clinical features were explored (Table 2). Patients who exhibited a negative PgR conversion were significantly more likely to have received postoperative endocrine therapy (p = 0.003). Patients who experienced a change in ER expression were less likely to have received postoperative radiation therapy, although this correlation was not significant (p = 0.052). No significant correlations were observed between a change in HER2 expression and any clinical factors. We then examined the potential correlations between smoking and clinical factors (Table 3). However, a significant correlation was only observed between a positive conversion of HER2 in recurrence and a history of smoking (p = 0.024). In addition, a univariate analysis with HER2 changes by pack-years showed that the odds ratio increased as pack-years increased (Table 4).

**Table 2:** Correlation between changes in receptor expression and clinical factors

Parameters	Estrogen receptor		*p* value	Progesterone receptor		*p* value	HER2		*p* value
	Negative conversion (*n* = 3)	The other (*n* = 47)		Negative Conversion (*n* = 15)	No change (*n* = 35)		Positive Conversion (*n* = 6)	No change (*n* = 44)	
Age at operation (years old)									
≤ 60	1 (33.3%)	25 (53.2%)	0.514	7 (46.7%)	19 (54.3%)	0.63	4 (66.7%)	22 (50.0%)	0.453
> 60	2 (66.7%)	22 (46.8%)		8 (53.3%)	16 (45.7%)		2 (33.3%)	22 (50.0%)	
Tumor size (mm)									
≤ 21.8	1 (33.3%)	24 (51.1%)	0.561	10 (66.7%)	15 (42.9%)	0.128	4 (66.7%)	21 (47.7%)	0.394
> 21.8	2 (66.7%)	23 (48.9%)		5 (33.3%)	20 (57.1%)		2 (33.3%)	23 (52.3%)	
Lymph node metastasis									
Negative	2 (66.7%)	41 (87.2%)	0.33	12 (80.0%)	31 (88.6%)	0.434	6 (100.0%)	37 (84.1%)	0.302
Positive	1 (33.3%)	6 (12.8%)		3 (20.0%)	4 (11.4%)		0 (0.0%)	7 (15.9%)	
Ki67 of primary tumor									
≤ 14%	2 (66.7%)	17 (36.2%)	0.301	5 (33.3%)	14 (40.0%)	0.664	1 (16.7%)	18 (40.9%)	0.26
> 14%	1 (33.3%)	30 (63.8%)		10 (66.7%)	21 (60.0%)		5 (83.3%)	26 (59.1%)	
Chemotherapy after surgery									
No	1 (33.3%)	36 (76.6%)	0.102	11 (73.3%)	26 (74.3%)	0.945	5 (83.3%)	32 (72.7%)	0.588
Yes	2 (66.7%)	11 (23.4%)		4 (26.7%)	9 (25.7%)		1 (16.7%)	12 (27.3%)	
Endocrine therapy after surgery									
No	1 (33.3%)	13 (27.7%)	0.836	0 (0.0%)	14 (40.0%)	0.003	2 (33.3%)	12 (27.3%)	0.762
Yes	2 (66.7%)	34 (72.3%)		15 (100.0%)	21 (60.0%)		4 (66.7%)	32 (72.7%)	
Radiation therapy after surgery									
No	1 (33.3%)	38 (80.9%)	0.056	10 (66.7%)	29 (82.9%)	0.213	5 (83.3%)	34 (77.3%)	0.743
Yes	2 (66.7%)	9 (19.1%)		5 (33.3%)	6 (17.1%)		1 (16.7%)	10 (22.7%)	
Trastuzumab after surgery									
No	3 (100.0%)	46 (97.9%)	0.804	15 (100.0%)	34 (97.1%)	0.518	6 (100.0%)	43 (97.7%)	0.716
Yes	0 (0.0%)	1 (2.1%)		0 (0.0%)	1 (2.9%)		0 (0.0%)	1 (2.3%)	
No-treatment after surgery									
No	3 (100.0%)	41 (87.2%)	0.519	15 (100.0%)	29 (82.9%)	0.091	5 (83.3%)	39 (88.6%)	0.715
Yes	0 (0.0%)	6 (12.8%)		0 (0.0%)	6 (17.1%)		1 (16.7%)	5 (11.4%)	
Age at recurrence (years old)									
≤ 62	1 (33.3%)	25 (53.2%)	0.514	8 (53.3%)	18 (51.4%)	0.904	4 (66.7%)	22 (50.0%)	0.454
> 62	2 (66.7%)	22 (46.8%)		7 (46.7%)	17 (48.6%)		2 (33.3%)	22 (50.0%)	
Recurrence site biopsied									
Local, regional lymph node	2 (66.7%)	43 (91.5%)	0.25	14 (93.3%)	31 (88.6%)	0.458	6 (100.0%)	39 (88.6%)	0.715
Distant metastasis	1 (33.3%)	4 (8.5%)		1 (6.7%)	4 (11.4%)		0 (0.0%)	5 (11.4%)	
Change of ER in recurrence									
Negative conversion	–	–	–	2 (13.3%)	1 (2.9%)	0.159	0 (0.0%)	3 (6.8%)	0.519
The other	–	–		13 (86.7%)	34 (97.1%)		6 (100.0%)	41 (93.2%)	
Change of PgR in recurrence									
Negative conversion	2 (66.7%)	13 (27.7%)	0.159	–	–	–	3 (50.0%)	12 (27.3%)	0.264
No change	1 (33.3%)	34 (72.3%)		–	–		3 (50.0%)	32 (72.7%)	
Change of HER2 in recurrence									
Positive conversion	0 (0.0%)	6 (12.8%)	0.519	3 (20.0%)	3 (8.6%)	0.264	–	–	–
No change	3 (100.0%)	41 (87.2%)		12 (80.0%)	34 (91.4%)		–	–	
Disease free survival									
≤ 792	2 (66.7%)	23 (48.9%)	0.561	8 (53.3%)	17 (48.6%)	0.764	3 (50.0%)	22 (50.0%)	1
> 792	1 (33.3%)	24 (51.1%)		7 (46.7%)	18 (51.4%)		3 (50.0%)	22 (50.0%)	

*HER2* human epidermal growth factor receptor 2, *ER* estrogen receptor, *PgR* progesterone receptor

**Table 3:** Correlation between smoking and clinical factors

Parameters	Smoking		*p* value
	No-smoker (*n* = 36)	Smoker (*n* = 14)	
Age at operation (years old)			
≤ 60	19 (52.8%)	7 (50.0%)	0.863
> 60	17 (47.2%)	7 (50.0%)	
Tumor size (mm)			
≤ 21.8	16 (44.4%)	9 (64.3%)	0.216
> 21.8	20 (55.6%)	5 (35.7%)	
Lymph node metastasis			
Negative	31 (86.1%)	12 (85.7%)	0.972
Positive	5 (13.9%)	2 (14.3%)	
Estrogen receptor (ER) of primary tumor			
Negative	10 (27.8%)	3 (21.4%)	0.654
Positive	26 (72.2%)	11 (78.6%)	
Progesterone receptor (PgR) of primary tumor			
Negative	12 (33.3%)	3 (21.4%)	0.420
Positive	24 (66.7%)	11 (78.6%)	
HER2 of primary tumor			
Negative	35 (97.2%)	13 (92.9%)	0.490
Positive	1 (2.8%)	1 (7.1%)	
Ki67 of primary tumor			
≤ 14%	15 (41.7%)	4 (28.6%)	0.402
> 14%	21 (58.3%)	10 (71.4%)	
Intrinsic subtype HRBC			
No	10 (27.8%)	2 (14.3%)	0.326
Yes	26 (72.2%)	12 (85.7%)	
Intrinsic subtype HER2BC			
No	35 (97.2%)	13 (92.9%)	0.490
Yes	1 (2.8%)	1 (7.1%)	
Intrinsic subtype TNBC			
No	27 (75.0%)	13 (92.9%)	0.163
Yes	9 (25.0%)	1 (7.1%)	
Chemotherapy after surgery			
No	26 (72.2%)	11 (78.6%)	0.654
Yes	10 (27.8%)	3 (21.4%)	
Endocrine therapy after surgery			
No	11 (30.6%)	3 (21.4%)	0.529
Yes	25 (69.4%)	11 (78.6%)	
Radiation therapy after surgery			
No	30 (83.3%)	9 (64.3%)	0.150
Yes	6 (16.7%)	5 (35.7%)	
Trastuzumab after surgery			
No	35 (97.2%)	14 (100.0%)	0.538
Yes	1 (2.8%)	0 (0.0%)	
No-treatment after surgery			
No	32 (88.9%)	12 (85.7%)	0.762
Yes	4 (11.1%)	2 (14.3%)	
Age at recurrence (years old)			
≤ 62	20 (55.6%)	6 (42.9%)	0.430
> 62	16 (44.4%)	8 (57.1%)	
Recurrence site biopsied			
Local, regional lymph node	31 (86.1%)	14 (100.0%)	0.108
Distant metastasis	5 (13.9%)	0 (0.0%)	
Change of ER in recurrence			
Negative conversion	2 (5.6%)	1 (7.1%)	0.836
The other	34 (94.4%)	13 (92.9%)	
Change of PgR in recurrence			
Negative conversion	9 (25.0%)	6 (42.9%)	0.224
No change	27 (75.0%)	8 (57.1%)	
Change of HER2 in recurrence			
Positive conversion	2 (5.6%)	4 (28.6%)	0.024
No change	34 (94.4%)	10 (71.4%)	
Change of intrinsic subtype in recurrent tumor			
No	32 (88.9%)	13 (92.9%)	0.682
Yes	4 (11.1%)	1 (7.1%)	
Disease free survival			
≤ 792	19 (52.8%)	6 (42.9%)	0.538
> 792	17 (47.2%)	8 (57.1%)	

*HER2* human epidermal growth factor receptor 2, *ER* estrogen receptor, *PgR* progesterone receptor

**Table 4:** Univariate analysis with positive conversion of HER2 in recurrence for smoking

Smoking	Change of HER2 in recurrence	Odd ratio	95% CI	*p* value
	No change/positive conversion			
No-smoker	34 (94.4%)/2 (5.6%)	Reference	Reference	
Pack-years of smoker				
≤ 25	3 (75.0%)/1 (25.0%)	5.667	0.390–82.237	0.243
25–50	6 (75.0%)/2 (25.0%)	5.667	0.664–48.333	0.124
> 50	1 (50.0%)/1 (50.0%)	17.000	0.753–383.892	0.096
Smoker	10 (71.4%)/4 (28.6%)	6.800	1.082–42.731	0.024

*HER2* human epidermal growth factor receptor 2, *CI* confidence intervals

### Prognostic analysis based on a change in receptor expression and smoking history

**Fig. 2:**
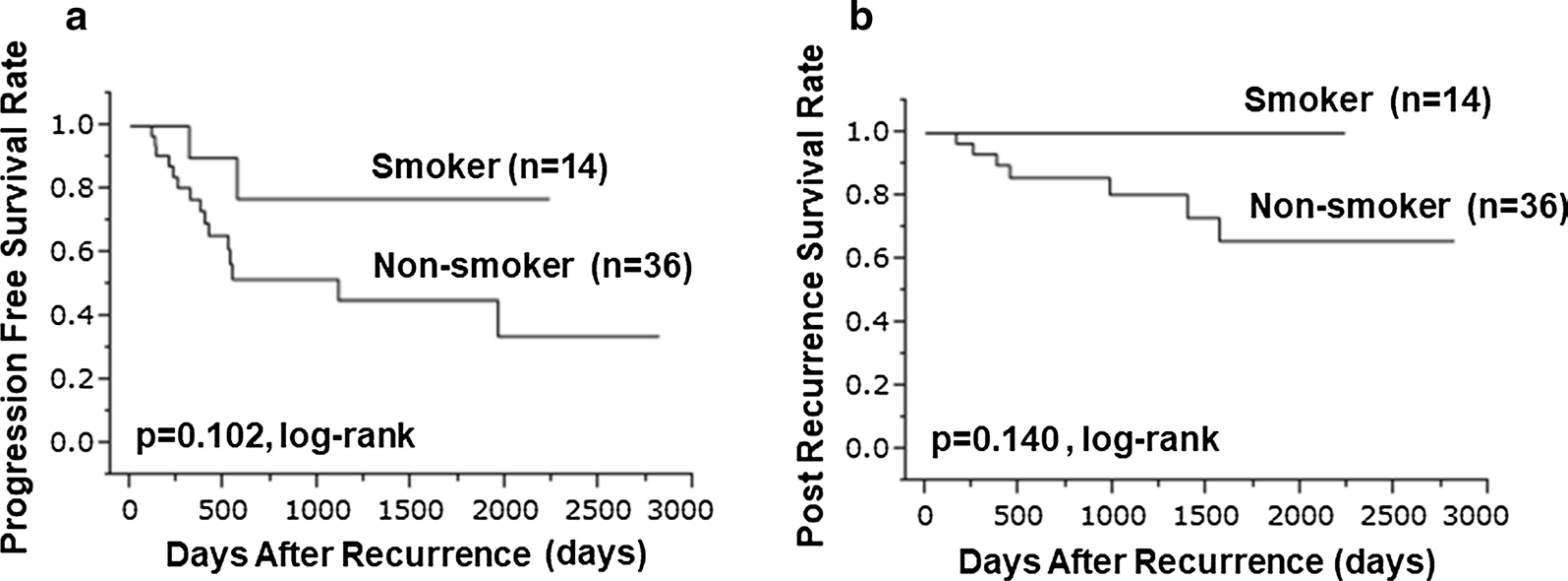
Regarding progression-free survival, there was no difference between smoker and non-smoker (p = 0.102, log-rank) (**a**). There was no difference in post-recurrence survival between smoker and non-smoker (p = 0.140, log-rank) (**b**)

The analysis revealed no significant difference in PFS between smokers and non-smokers (p = 0.102, log-rank; Fig. 2a). A univariate analysis identified significant correlations of chemotherapy after surgery and a change in intrinsic subtype in the recurrent tumor with a poor PFS (p = 0.015, HR = 3.734, 95% CI 1.316–10.115 and p = 0.039, HR = 3.889, 95% CI 1.083–11.236, respectively) (Table 5). However, no factors independently associated with PFS were identified in a multivariate analysis.

**Table 5:** Univariate and multivariate analysis with progression-free survival

Parameters	Univarite analysis			Multivarite analysis		
	Hazard ratio	95% CI	*p* value	Hazard ratio	95% CI	*p* value
Age at operation (years old)						
≤ 60 vs > 60	1.871	0.716–5.172	0.201			
Tumor size (cm)						
≤ 21.8 vs > 21.8	1.708	0.654–4.714	0.274			
Lymph node metastasis						
Negative vs positive	2.503	0.794–6.779	0.110			
Estrogen receptor (ER) of primary tumor						
Negative vs positive	0.753	0.264–2.690	0.631			
Progesterone receptor (PgR) of primary tumor						
Negative vs positive	0.296	0.555–1.765	0.296			
HER2 of primary tumor						
Negative vs positive	3.571	0.190–20.270	0.310			
Ki67 of primary tumor						
≤ 14% vs > 14%	0.726	0.276–2.017	0.525			
Intrinsic subtype HRBC						
No vs Yes	0.574	0.198–2.068	0.363			
Intrinsic subtype HER2BC						
No vs yes	3.571	0.190–20.270	0.310			
Intrinsic subtype TNBC						
No vs yes	1.394	0.318–4.345	0.618			
Chemotherapy after surgery						
No/yes	3.734	1.316–10.115	0.015	2.953	0.844–9.775	0.088
Endocrine therapy after surgery						
No vs yes	0.650	0.240–2.049	0.435			
Radiation therapy after surgery						
No vs yes	1.352	0.429–3.654	0.581			
Trastuzumab after surgery						
No vs yes	13.762	0.680–107.629	0.077	7.081	0.323–67.456	0.172
No-treatment after surgery						
No vs yes	0.502	0.028–2.486	0.461			
Age at recurrence (years old)						
≤ 62 vs > 62	1.690	0.639–4.557	0.287			
Recurrence site biopsied						
Local, regional lymph node vs distant metastasis	2.225	0.347–8.089	0.340			
Change of ER in recurrence						
The other vs negative conversion	3.228	0.503–11.746	0.182			
Change of PgR in recurrence						
No change vs negative conversion	1.070	0.383–2.827	0.892			
Change of HER2 in recurrence						
No change vs positive conversion	1.423	0.324–4.485	0.599			
Change of intrinsic subtype in recurrent tumor						
No vs yes	3.889	1.083–11.236	0.039	3.645	0.910–12.622	0.066
Disease free survival						
≤ 792 vs > 792	0.697	0.250–1.852	0.469			
Smoker						
No vs yes	0.312	0.049–1.109	0.075	0.273	0.042–1.003	0.051

*CI* confidence intervals, *HER2* human epidermal growth factor receptor 2, *HRBC* hormone receptor positive breast cancer, *HER2BC* human epidermal growth factor receptor 2-enriched breast cancer, *TNBC* triple negative breast cancer

Similarly, no significant difference in PRS was identified between smokers and non-smokers (p = 0.140, log-rank; Fig. 2b). Although a univariate analysis identified a biopsied distant metastasis as associated significantly with a poor PRS (p = 0.041, HR = 8.527, 95% CI 1.114–52.010), no significant independent factors were identified in a multivariate analysis (Table 6). In summary, our results do not suggest an association between smoking and the prognosis after relapse.

**Table 6:** Univariate and multivariate analysis with post-recurrence survival

Parameters	Univarite analysis			Multivarite analysis		
	Hazard ratio	95% CI	*p* value	Hazard ratio	95% CI	*p* value
Age at operation (years old)						
≤ 60 vs > 60	0.878	0.173–3.993	0.865			
Tumor size (cm)						
≤ 21.8 vs > 21.8	1.464	0.322–7.445	0.616			
Lymph node metastasis						
Negative vs positive	1.619	0.230–7.587	0.581			
Estrogen receptor (ER) of primary tumor						
Negative vs positive	0.326	0.072–1.661	0.164			
Progesterone receptor (PgR) of primary tumor						
Negative vs positive	0.341	0.075–1.748	0.182			
HER2 of primary tumor						
Negative vs positive	8.234	0.405–65.239	0.136			
Ki67 of primary tumor						
≤14% vs > 14%	0.702	0.154–3.581	0.649			
Intrinsic subtype HRBC						
No vs yes	0.261	0.057–1.328	0.100	0.691	0.093–5.986	0.718
Intrinsic subtype HER2BC						
No vs yes	8.234	0.405–65.239	0.136			
Intrinsic subtype TNBC						
No vs Yes	2.423	0.347–11.289	0.325			
Chemotherapy after surgery						
No/yes	4.133	0.910–20.999	0.065	3.692	0.537–24.657	0.176
Endocrine therapy after surgery						
No vs yes	0.770	0.165–5.403	0.760			
Radiation therapy after surgery						
No vs yes	2.279	0.448–10.372	0.297			
Trastuzumab after surgery						
No vs yes	11.924	0.589–93.487	0.090	0.552	0.014–23.153	0.735
No-treatment after surgery						
No vs yes	–	–	0.245			
Age at recurrence (years old)						
≤ 62 vs > 62	1.008	0.197–4.621	0.992			
Recurrence site biopsied						
Local, regional lymph node vs Distant metastasis	8.527	1.114–52.010	0.041	6.962	0.312–73.359	0.178
Change of ER in recurrence						
The other vs negative conversion	2.194	0.116–12.967	0.509			
Change of PgR in recurrence						
No change vs negative conversion	0.583	0.083–2.742	0.509			
Change of HER2 in recurrence						
No change vs positive conversion	–	–	0.203			
Change of intrinsic subtype in recurrent tumor						
No vs yes	1.194	0.063–7.080	0.873			
Disease free survival						
≤ 792 vs > 792	0.936	0.177–4.486	0.934			
Smoker						
No vs Yes	–	–	0.052	–	–	0.118

*CI* confidence intervals, *HER2* human epidermal growth factor receptor 2, *HRBC* hormone receptor positive breast cancer, *HER2BC* human epidermal growth factor receptor 2-enriched breast cancer, *TNBC* triple negative breast cancer

## Discussion

Decisions regarding the selection of breast cancer therapies require an accurate determination of the ER, PgR, and HER2 expression status of the tumor, which are usually determined via biopsy to achieve a definitive diagnosis. However, a biopsy specimen represents only part of lesion and often differs from the surgical specimen. Previous reports describe ER expression concordance rates between biopsy specimens and surgical specimens as high as 92–98% and similarly high PgR concordance rates of 85–97% [13, 14]. However, the reported HER2 concordance rates are slightly lower, at 80–90% [13, 15, 16]. Moreover, anticancer therapy affects the expression of these receptors. In a meta-analysis of patients who underwent neoadjuvant chemotherapy for breast cancer, ER and PgR discordance rates of 2.5–17% and 5.9–51.7%, respectively, were reported [17]. There are reports that it turns out to be often positive, while others report that it often turns negative. Regarding HER2, studies reported discordance rates between biopsy specimens and surgical specimens of 1.3–20% in patients who received neo-adjuvant chemotherapy (NAC) without trastuzumab and of 12–43% in whose who received NAC with trastuzumab. These data suggest that trastuzumab therapy induces a negative HER2 conversion. In our study, therefore, we targeted cases that were preoperative treatment-naïve to address the potential differences between biopsy and surgical specimens and changes due to NAC.

Some reports have described differences in the patterns of receptor expression between surgical specimens and recurrent tumor specimens [7, 8, 18–20]. A change in the ER status is observed in approximately 15% of cases, and the numbers of cases with increasing and decreasing expression are roughly equivalent. In contrast, a change in the PgR status is observed in approximately 25–40% of cases, and usually involves decreased expression. Changes in HER2 are observed in approximately 10% of cases, and more frequently tend to involve decreased expression. Consequently, some reports describe a change in breast cancer subtype to TNBC in recurrence, and these cases tend to have a worse prognosis than those with primary TNBC [7, 8]. In our study, we also compared the receptor expression patterns between surgical specimen and corresponding biopsies of recurrent tumors, which involved the local or regional lymph nodes in 90% of cases. The primary tumor type was HRBC in 76.0% of cases, and the frequencies of change in the ER, PgR, and HER2 statuses between the surgical and recurrent specimens were similar to those in previous reports.

In vitro experiments have demonstrated the ability of tobacco components to increase the proliferative capacity and induce malignant transformation in breast cancer cells [4–6], and various reports have described an association between ER expression and smoking in clinical practice [21–26]. However, few reports have explored the potential relationship between HER2 expression and smoking in breast cancer. Notably, we observed a significant correlation between smoking and a positive conversion of HER2 in our study. Although smoking is a known etiologic factor in lung cancer, an interesting potential correlation between HER2 mutation and lung cancer in never-smokers has attracted clinical attention [27, 28]. However, in vitro experiments have demonstrated the ability of tobacco components to induce HER2 [29] and amplify the expression of EGFR and HER3 [29, 30]. Crosstalk has been identified within the HER family, and potentially the amplification of another HER family member may enhance the expression of HER2 [31, 32]. In the future, it is necessary to examine the biological changes caused by tobacco components in breast cancer cells using immunohistochemical staining, genetic analysis, and protein quantification in vitro.

The choice of treatment after recurrence varied among the cases in our study, as some patients underwent excision of the recurrent lesions and others began anticancer therapy. Consequently, an evaluation of prognosis was challenging. However, we found that a negative hormone receptor conversion, positive HER2 conversion, and change of the intrinsic breast cancer subtype appears to reduce the DFS. However, smokers in our study appeared to have a better DFS and OS, possibly because the switch from ER+/HER2− to ER+/HER2+ breast cancer in most smokers enabled the administration of more effective drug treatment.

This study had a few limitations of note. Particularly, we only obtained data about the smoking history up to surgery, and the use of an interview to collect these data may have introduced bias. Although we agree that the postoperative smoking status is important, some reports suggest that the total smoking history is more important than the current smoking status with respect to carcinogenesis and recurrence [33, 34]. Moreover, we were not able to reach clear conclusions about receptor expression patterns on distant metastases, as most recurrences occurred in local or regional lymph nodes. However, the identification of a correlation between smoking and the positive conversion of HER2 at recurrence suggests that appropriate treatment may not have been administered to patients with distant metastases. We must therefore consider the possible link between smoking and HER2 amplification when evaluating cases in which a biopsy of a distant metastasis cannot be performed.

## Conclusions

In conclusion, our results emphasize that biological changes during breast cancer recurrence should receive careful clinical consideration because of the potential effects on treatment after recurrence. However, smoking only appeared to have an effect on HER2 expression patterns after recurrence, but not on survival prognosis.

## Supplementary information

**Additional file 1: Table S1.** The recurrence cases of organs that was not biopsied at the simultaneously.

## Data Availability

The datasets supporting the conclusions of this article are included within the article.

## Abbreviations

CI: Confidence interval
CT: Computed tomography
ER: Estrogen receptor
HRBC: Hormone receptor-positive breast cancer
HER2: Human epidermal growth factor receptor 2
HER2BC: HER2-enriched breast cancer
HR: Hazard ratio
NAC: Neo-adjuvant chemotherapy
PFS: Progression-free survival
PgR: Progesterone receptor
PRS: Post-recurrence survival
REMARK: Reporting Recommendations for Tumor Marker Prognostic Studies
TNBC: Triple-negative breast cancer
US: Ultrasonography
