# Supplementary material for: The effect of smoking on biological change of recurrent breast cancer
Source: J Transl Med. 2020 Apr 5;18:153. doi: 10.1186/s12967-020-02307-x (PMC7132886; doi:10.1186/s12967-020-02307-x)
Supplement: Supplementary file 1 — Additional file 1: Table S1. The recurrence cases of organs that was not biopsied at the simultaneously. [file 12967_2020_2307_MOESM1_ESM.docx]

**Additional file 1; Table S1. The recurrence cases of organs that was not biopsied at the simultaneously.**

| Recurrent tumor site biopsied | Synchronous recurrence site |
| --- | --- |
| Local | One case; regional lymph node, one case; lung, one case; bone |
| Regional lymph node | Four cases; lung |
